# Adsorptive Decolorization of a Disodium Terephthalate Solution from Monomer Recycling of Polyester

**DOI:** 10.3390/polym18030345

**Published:** 2026-01-28

**Authors:** Charlotte Lücking, Mandy Paschetag, Stephan Scholl

**Affiliations:** 1 Institute for Chemical and Thermal Process Engineering, Technische Universität Braunschweig, Langer Kamp 7, 38106 Braunschweig, Germany; charlotte.luecking@tu-braunschweig.de (C.L.); m.paschetag@tu-braunschweig.de (M.P.); 2 matterr GmbH, Friedrich-Seele-Straße 3, 38122 Braunschweig, Germany

**Keywords:** chemical recycling, alkaline hydrolysis, activated carbon, terephthalic acid, circular economy, depolymerization

## Abstract

The global economy is increasingly faced with the challenge of accepting its responsibility for recycling polyester textile waste. With back-to-monomer recycling technologies, PET can be recycled to its monomers, terephthalic acid and ethylene glycol. The recycling of polyester-containing textiles requires the complete separation of all contaminating materials, dyes, and additives, which can only be achieved by depolymerization technologies. This article presents the adsorptive decolorization of a disodium terephthalate solution from the alkaline hydrolysis of polyester textile waste. The influence of different adsorbents, temperature (30–80 °C), and pH value (7–12) on the adsorptive decolorization process is investigated. As a result, activated carbons for decolorization have been identified. It was found that the adsorption process is favorable at neutral pH and a temperature of 80 °C. The findings show that a color value within the industrial specification can be obtained for recycled terephthalic acid using activated carbon adsorption. This adds a key step for high-quality textile-to-textile recycling and thus contributes to a circular economy for polyester.

## 1. Introduction

The continuous growth of textile fiber production presents a significant challenge to the global economy. In 2023, the annual fiber production increased to a record of 124 million tonnes, with polyester being the most widely used fiber accounting for a market share of 57% of total production volume. In fiber production, polyethylene terephthalate (PET) is the most important polyester, resulting in an annual production volume of 71 million tonnes [1,2]. The persistent increase in production volumes is associated with substantial consumption of critical resources, including water, energy, and fossil-based raw materials, as well as the generation of large amounts of production waste and emissions throughout the manufacturing cycle [3]. Polyester fibers are substantial for a wide range of applications, like clothing, home textiles, and technical textiles; however, they often used with a short lifetime.

The resulting constant growth of polyester-containing textile waste emphasizes the necessity of sustainable recycling technologies. To date, only 12.5% of the annual production volume is generated from recycled PET, being primarily derived from plastic bottles by up to 98% [1]. This reuse of textiles, which are often heavily colored or mixed with other materials and additives, represents a downcycling of high-quality PET products approved for food contact [4]. Post-production and post-consumer recycled textiles represent less than 1% of the global fiber market [1]. Meanwhile, around 73% of the polyester produced worldwide is lost to the material cycle through landfilling or incineration [5].

At present, no economically established large-scale recycling technologies are available that ensure a quality-preserving closed-loop solution for the recycling of polyester-containing textiles [4]. The major challenge in textile recycling is the complete removal of dyes and additives to obtain high-purity recyclates that enable textile-to-textile recycling without compromising quality [6].

### Recycling Pathways for Polyester-Containing Textile Waste

To date, textile recycling of PET primarily relies on mechanical processes. Overall, as long as the available feedstock quality is sufficient to achieve the desired product quality, mechanical recycling processes represent the most economically and environmentally advantageous recycling route. In mechanical recycling, the primary objective is to break down the textile structure into individual fibers while minimizing fiber damage. The recovered fibers can subsequently be respun into yarns, often by adding virgin fibers to improve spinnability and product quality, or reused in the production of nonwoven fabrics, like painter’s fleece or insulation materials [7]. However, these processes reach a limit when it comes to the recycling of mixed or colored feed materials [4,8,9]. Accordingly, the textiles are pre-sorted by color, cleaned and cut into smaller pieces to improve processing, and after which the recovered fibers are spun into recycled yarn [10]. As a result, chemicals such as dyes, finishes, and additives, e.g., coatings, flame retards induced during production, cannot be removed during mechanical processes [5].

To broaden the spectrum of processable waste streams, further recycling processes including thermo-mechanical and thermo-chemical recycling, solvent-based separation, as well as depolymerization technologies are investigated [4,11,12,13].

In thermo-mechanical recycling, thermoplastic textiles such as polyesters or polyamides are remelted and processed into granules or new fibers, while the molecular structure of the polymer is retained [4]. Typically, only well-defined, uncontaminated production waste is used and the addition of virgin polymer is often required to obtain reusable end products. As with mechanical recycling, dyes and additives remain in the recycled material, meaning color and quality of the output are determined by the feedstock material [9].

In contrast, solvent-based separation describes the specific dissolution of a target polymer from mixed waste using a selective solvent. This process is a physical method preserving the molecular structure of the polymer. Although surface coatings and dyes can be separated during the recycling, the usage of hazardous solvents is often necessary, also bearing the risk of solvent residues remaining in the recyclate [14].

Thermo-chemical recycling includes pyrolysis and gasification. In these processes, heat is used with or without a partial oxidation reaction, to convert polymers into low-molecular-weight components, such as pyrolysis oils or synthesis gas. These recycling options do not allow textile-to-textile recycling, since the products are not relevant for PET fiber production, but can be reused in the (petro-)chemical industry [4]. In addition, these recycling routes mainly target polyolefin-based feedstocks, with only minor PET content being feasible [15].

In contrast, depolymerization technologies include hydrolysis, methanolysis, glycolysis, and biochemical processes using enzymes. The various technologies differ in terms of reactants, reaction conditions, such as temperature, pressure, reaction time, and use of catalysts, and the obtained products. Depending on the depolymerization technology, main products include monomers or intermediate products of PET production such as terephthalic acid (TA), ethylene glycol, dimethyl terephthalate (DMT), or bis(2-hydroxyethyl)terephthalate (BHET) [16,17,18,19].

By resolving the PET molecular structure, the complete separation of dyes and additives is enabled, which allows the recovery of monomers in fossil-like quality so that they can be used for the production of new PET without any loss of quality [20,21]. Therefore, the removal of dyes is a crucial step. PET in textiles is almost exclusively dyed with disperse dyes. Chemically, these dyes can be classified into 50% of azo compounds, about 25% anthraquinones, and the rest are methine, nitro, and naphthoquinone dyes [22]. These dyes must be separated during the respective recycling processes to obtain pure recyclates.

Another challenge in recycling mixed waste fractions results from the generally unknown composition of the waste [23] and therefore dye mixtures, which require an encompassing decolorization process. Often, adsorptive processes using adsorbents such as activated carbons are employed to remove residual impurities, mostly colored compounds [24,25,26,27].

Overall, chemical recycling technologies complement mechanical recycling by enabling the processing of dyed and mixed textile waste streams that cannot be recycled mechanically. However, chemical recycling processes require higher energy input and the use of chemical reagents, thus increasing the required recycling effort. In terms of alkaline hydrolysis of PET, the recovered monomers TA and ethylene glycol are chemically identical to those used in the production of fossil-based PET and can therefore be directly integrated into existing polymerization plants. Nevertheless, using chemical reagents also poses a risk of associated degradation of foreign fiber materials. Consequently, recycling fiber blends, particularly those containing polyamides or elastane, requires further detailed investigation to enable the effective separation of potential undesired by-products, which in turn necessitate comprehensive purification of the recovered monomers [7].

This work builds on the depolymerization of PET from textile waste by alkaline hydrolysis, resulting in the monomers TA and ethylene glycol. It is first shown that adsorptive decolorization can be used to recover recycled terephthalic acid (rTA) that is in accordance with industrial specifications in terms of color value. This study systematically investigates the effect of adsorbent, temperature, and pH on the decolorization of a disodium terephthalate solution from polyester textile waste. For the first time, optimized adsorption parameters that ensure specification-compliant monomer recycling are reported in this article. For the monomer TA, a color value below 10 H° is one of the critical quality attributes [28]. The platinum–cobalt color number (Pt-Co color number), also known as the Hazen color number or APHA color number, allows the color of clear liquids to be determined in Pt-Co units by measuring light transmission. The color of the Pt-Co standard ranges from light yellow to brown [29]. With regard to the LAB color scale, a b-value of less than two is required to meet the specification. The LAB scale describes the three-dimensional color space, whereby the L-value indicates brightness, the b-value indicates the shift from blue to yellow and the a-value specifies the color on the red to green axis [30].

## 2. Materials and Methods

### 2.1. Textile Sample Material

The decolorization experiments were conducted using the product solution obtained after depolymerization. For experimental investigations, a post-production technical textile with a defined polyester content of 100% was utilized to receive homogeneous sample material. The specific dye types employed for coloration of the material are unknown. Therefore, information on their molecular structures and surface charge characteristics is not available. In preparation for depolymerization, the textiles were first shred into fibers, which were then compressed into pellets.

### 2.2. Reagents

For the decolorization experiments, different activated carbons as well as the polymeric resin Amberlite XAD-7HP, supplied by Merck KGaA, Darmstadt, Germany, were used as adsorbents. The characteristics of activated carbons are displayed in Table 1. All activated carbons were supplied in granular form and therefore grinded to powder prior to testing, to enhance surface accessibility, reduce diffusion limitations, and ensure comparability independent of particle size. Gentle milling primarily affects the particle size distribution and consequently the external particle surface, while leaving the internal pore structure intact. Since the external surface represents only a small proportion of the total pore surface area, changes due to grinding can be considered negligible. In addition, a change in surface charge is not expected.

Sulfuric acid (H_2_SO_4_) (2 wt% and 96 wt%, p.a.) for pH adjustment and precipitation as well as ammonia solution (25 wt%, p.a.) for UV/VIS analytics and sodium hydroxide (NaOH) (purity ≥ 98%, solid pearls with diameter < 3 mm) for depolymerization were supplied by Carl Roth GmbH & Co., KG, Karlsruhe, Germany.

### 2.3. Depolymerization of Polyester Textiles

**Figure 1:**
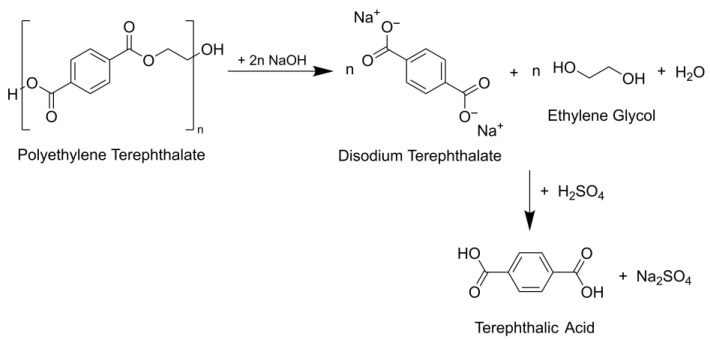
Reaction scheme of PET depolymerization by alkaline hydrolysis.

The depolymerization using alkaline hydrolysis follows the reaction scheme shown in Figure 1.

The process concept and experimental setup for the depolymerization of PET containing waste samples were first described in Biermann et al. [31]. The textile samples are depolymerized by alkaline hydrolysis using a twin screw extruder (ZSE 27 MAXX, Leistritz Extrusionstechnik GmbH, Nürnberg, Germany). For depolymerization of textile material, the samples are shredded and compacted to obtain a dosable feed. Then, 7 kg h^−1^ PET waste and 3 kg h^−1^ of NaOH are processed at a stoichiometric ratio of 2.1 mol NaOH mol PET^−1^. Compared to Figure 1, a slight excess of NaOH is used to ensure a complete PET conversion. In the extruder, the components are mixed and kneaded at a process temperature of up to 160 °C. After about 75% of the extruder length, the temperature is lowered to 80 °C by the addition of 10 kg h^−1^ water. The product mixture is completely dissolved in water and undissolved components within the solution are removed by filtration to obtain a particle-free product solution used for the following experiments. Besides water, ethylene glycol, and unreacted PET, the product solution contains the intermediate product disodium terephthalate, from which rTA is precipitated by adding H_2_SO_4_. As a by-product, sodium sulfate (Na_2_SO_4_) is formed. To recover the rTA with required purity, the product solution must be purified. This can be achieved by means of adsorptive decolorization of the disodium terephthalate solution.

### 2.4. Investigation of the Impact of pH and Temperature on Adsorptive Decolorization

The product solution obtained from the PET textile sample described in Section 2.1 was employed for the experimental investigation of pH and temperature effects on adsorptive decolorization. The solution was obtained by depolymerization using alkaline hydrolysis, as detailed in Section 2.3. For each experiment, 100 mL of the product solution were transferred to a flask. Subsequently, 50 g L^−1^ of the activated carbon AC-2 were added. The batch was stirred for 24 h until equilibrium, indicated by no further reduction in color value as measured by UV/VIS spectroscopy. At the end of the experiment, the activated carbon was filtered off using a filter with a mesh of 2–4 µm. The rTA was precipitated from the filtered solution by adding H_2_SO_4_ (96 wt%). Afterwards, the rTA was filtered from the solution, washed with deionized water, and dried at 80 °C until mass constancy. The rTA color value was determined according to Section 2.6. For the experiments on pH investigation, the pH of the solution was adjusted using H_2_SO_4_ (25 wt%) prior to the addition of activated carbon. To only generate Na_2_SO_4_ as a by-product, the same acid is employed for pH adjustment as well as precipitation. The experiments were carried out at 80 °C. In contrast, for temperature variation experiments, the pH was kept constant at pH 7. The tests were performed as a triplicate. In addition, a blind sample was analyzed.

### 2.5. Investigation of the Impact of Adsorbent on Adsorptive Decolorization

Batch experiments were conducted to identify suitable adsorbents for decolorization of the product solution obtained from a PET textile after depolymerization. The experiments were carried out according to the following procedure. For each experiment, 100 mL of the product solution was transferred to a flask. Subsequently, the pH of the solution was adjusted to pH 7 by adding H_2_SO_4_ (25 wt%). Then, 50 g L^−1^ of activated carbon were added. The tests were performed as a triplicate for each activated carbon mentioned in Section 2.2. The batch was stirred for 24 h at 80 °C until equilibrium. After completion of the test, the activated carbon was filtered from the solution and the rTA was recovered and analyzed as described above.

### 2.6. Analysis of Color Value

The color value of the rTA was determined by UV/VIS spectroscopy, employing a SPECORD 50 from Jena Analytic AG, Jena, Germany. Therefore, a quantity of 50 mg of rTA was dissolved in 10 mL of ammonia solution (2 M) at ambient temperature to achieve high solubility. To analyze the solution, the absorbance was measured between 230 nm and 900 nm. The color value in Hazen and the LAB values was exported using the ASpect UV 1.5 software.

## 3. Results and Discussions

### 3.1. Influence of pH on Adsorptive Decolorization

**Figure 2:**
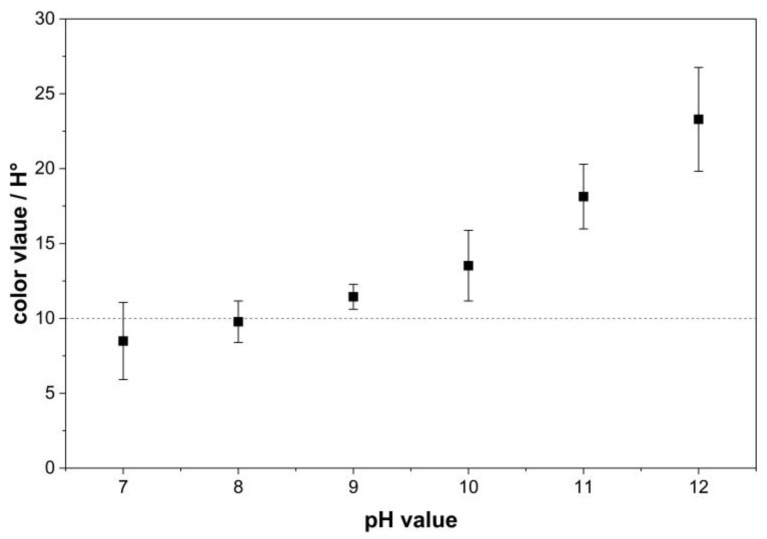
Influence of product solution pH on the color value of rTA. Means and standard deviations are shown. The dotted line indicates the specification limit for the rTA color value of 10 H°.

The pH value of aqueous solutions has been identified as a pivotal factor of adsorption processes by affecting the surface charge of the adsorbent as well as the ionization of the adsorbate [32]. Figure 2 shows the results of the conducted adsorption experiments. The graph indicates a substantial decrease in the color value of the rTA as the pH value decreases. Similar behavior is expected for all activated carbons listed in Table 1. Therefore, the results for activated carbon AC-2 are shown as a representative example. At an alkaline pH of 12, a color value of 23.3 H° was obtained, whereas at a neutral pH of 7, the color value decreased to 8.5 H°. To achieve the target color value for rTA of <10 H°, the pH of the solution must therefore be adjusted to below pH 8. This results in a color value of 9.5 H°.

The neutral pH of the product solution leads to a positive influence on the adsorption process, as the surface of activated carbon is charged nearly electrically neutral under these conditions. Similarly, disperse dyes are largely uncharged at a pH of 7. This results in a reduced electrostatic repulsion between the dye molecules and the activated carbon surface. At the same time, hydrophobic as well as π–π interactions become dominant, promoting strong adsorption onto the activated carbon, which possesses a predominantly non-polar and hydrophobic surface.

At alkaline pH values, the surface of the activated carbon becomes negatively charged due to deprotonation. Under these conditions, the dye molecules may also acquire an anionic character. Consequently, an enhanced electrostatic repulsion occurs between the negatively charged activated carbon surface and the dye molecules, resulting in reduced adsorption. Adsorption under acidic conditions below pH 7 is not feasible, as disodium terephthalate precipitates and rTA is formed. Additional studies, including determination of the point of zero charge or surface analysis of the activated carbon, would allow a more detailed investigation of the adsorption mechanism and should be considered for future experiments.

Numerous other examples of the influence of pH value on the adsorption of textile dyes can be found in the literature. Optimal adsorption conditions vary among different dye systems and adsorbents. Nevertheless, a neutral pH of 7has been reported to provide the most favorable adsorption performance in various applications, which is in line with the presented findings [33].

Guillén–Mallette et al. determined the point of zero charge of activated carbons, indicating the pH value at which the carbon surface has no net charge. This point of zero charge of carbons activated by phosphoric acid values of pH from 7.6 to 7.98 indicates slightly anionic and nearly neutral surface properties [34].

Chowdhury and Saha investigated the influence of pH on the adsorption behavior of malachite green using alkali-treated fly ash. The study revealed that adsorption was most effective at neutral pH of 7 [35]. At lower pH values, protonation of the dye molecules reduces adsorption efficiency due to electrostatic repulsion and competition between protons and dye molecules for active binding sites. An increase in the solution pH leads to deprotonation of the dye molecules, resulting in a higher negative surface charge on the adsorbent and consequently more efficient dye removal [36].

Gupta et al. examined the effect of pH variation on the adsorption behavior of carbon slurry. Their results showed that under acidic conditions, strong electrostatic repulsion between the positively charged adsorbent surface and dye molecules significantly reduced adsorption, particularly at pH values below 6.2. As the pH increased to neutral, adsorption improved markedly due to enhanced electrostatic repulsion between the dye molecules and adsorbent surface. However, a further increase in pH beyond neutrality resulted in a decline in adsorption performance [37].

### 3.2. Influence of Temperature on Adsorptive Decolorization

**Figure 3:**
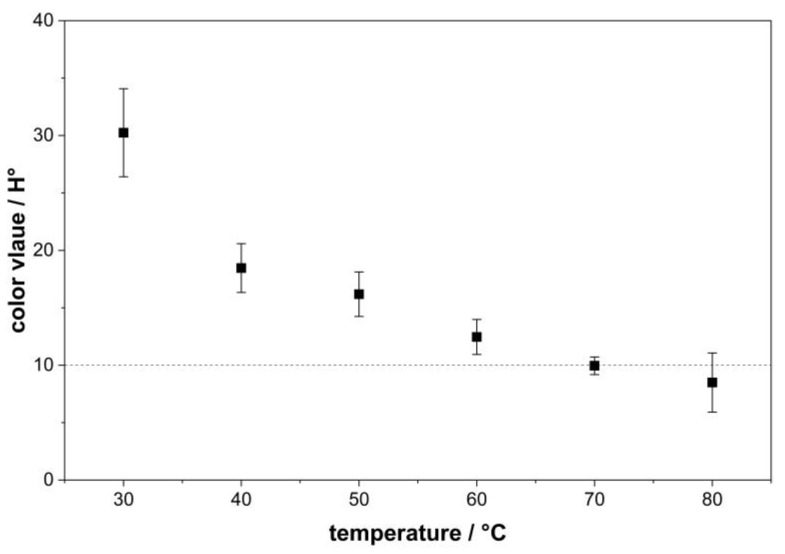
Influence of product solution temperature on the color value of rTA. Means and standard deviations are shown. The dotted line indicates the specification limit for the rTA color value of 10 H°.

Next to the influence of pH, the adsorption process in dye removal is also affected by temperature. Figure 3 presents the results of the experiments, investigating the effect of temperature on decolorization. The graph shows a decreasing color value of rTA with increasing adsorption temperature. Similar behavior is expected for all activated carbons listed in Table 1. Therefore, the results for activated carbon AC-2 are shown as a representative example. At an adsorption temperature of 30 °C, a color value of 30.2 H° was obtained, while at higher temperatures of 80 °C, the value decreased to 8.5 H°. To achieve the target color value for rTA of below 10 H°, a minimum adsorption temperature of 70 °C is required, at which a color value of 9.9 H° can be observed.

According to Foo and Hameed, the temperature has two major effects on the adsorption process. An increase in temperature is considered to enhance the diffusion rate of adsorbate molecules across the external boundary layer and into the internal pores of the adsorbent particles, primarily due to the reduced viscosity of the solution. Moreover, temperature can shift the equilibrium of the adsorption process between the adsorbent and the adsorbate [28,38]. Generally, the adsorption equilibrium of adsorption processes is known to decrease as temperature rises. The resulting decrease in adsorption capacity is primarily due to an enhanced desorption step and to weaker physical interactions between the active sites of activated carbon and the dye molecules. At the same time, higher temperatures increase the kinetic energy of the dye molecules, resulting in a higher collision frequency between the adsorbent and the adsorbate. Therefore, temperature affects not only the process of adsorption, but also desorption and the reversibility of adsorption equilibrium [38].

The results of this study regarding the influence of temperature do not fully align with the general theoretical considerations for adsorption processes but correspond with previously published data for comparable systems. Nevertheless, differences in application as well as investigated dye types and adsorbents examined in these studies should be taken into account. Further investigations are required to fully describe the adsorption mechanism present in this study.

Gerçel et al. reported that the equilibrium adsorption capacity of Disperse Orange 25 on activated carbon derived from *E. rigida* increased as the temperature increased from 10 °C to 20 °C. It is indicated by the authors that the adsorption process is thermodynamically favored at higher temperatures [39].

In a comparable study, Bulut et al. reported that the adsorption of Direct Blue 71 onto wheat shells became more efficient at higher temperatures up to 40 °C [40].

Moreover, Watts investigated the decolorization of imidazole-based ionic liquids using both polar and non-polar adsorbents, with activated carbons showing the most favorable performance. The study was conducted over a temperature range of 30 to 90 °C, revealing that the decolorization kinetics increased with temperature and that the highest degree of decolorization was achieved at 90 °C. Watts attributed these findings to a physical adsorption mechanism, identifying thermal aging and the enhanced diffusion of chromophores as the main factors for the temperature dependence of the adsorption process [41].

### 3.3. Influence of the Adsorbent

**Figure 4:**
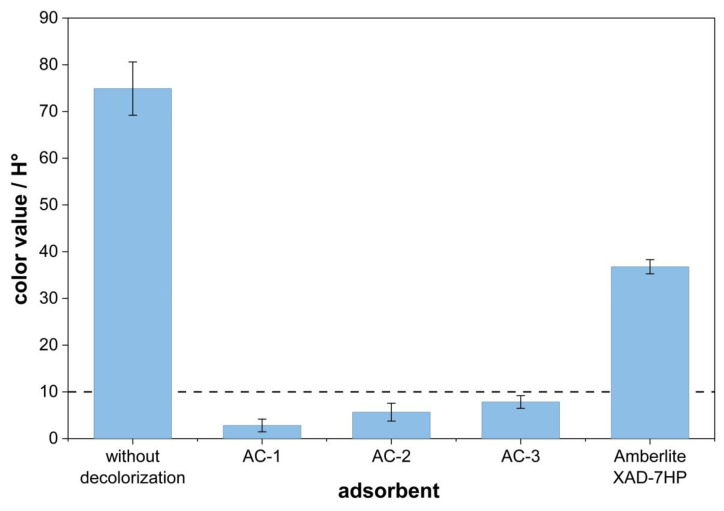
Influence of the adsorbent on decolorization of a disodium terephthalate solution. Means and standard deviations are shown. The dotted line indicates the specification limit for the rTA color value of 10 H°.

The influence of different adsorbents was investigated in accordance with the experimental description in 2.5. Figure 4 shows the results of the color value in Hazen after decolorization of the product solution with various adsorbents. Without decolorization, the product solution has a color value of 75 H°. The results show that all activated carbons, AC-1, AC-2, and AC-3, reduce the color value of the product solution to below 10 H°, thereby meeting the color specification of fossil-based TA. The best performance is achieved with AC-1, yielding a rTA color value of 2.8 H° after adsorption, whereas the use of AC-2 results in a color value of 5.7 H° and application of AC-3 achieves a color value of 7.8 H°. By contrast, the use of the polar polymer resin Amberlite XAD-7HP does not meet the TA specification, reducing the color value only to 36.8 H°.

The analysis of the LAB color values shows consistent results. Table 2 presents the results of the LAB color value measurements after decolorization using the respective adsorbent.

The results of the color values show that all activated carbons can meet the color value specification for the LAB values. Compared to fossil TA produced from crude oil, very high L-values of >99 are achieved by means of adsorptive purification. The L-value indicates the brightness, with a high value of maximum 100 representing a white color. The best results are obtained for the activated carbon AC-1. The b-value relevant for the specification results in 0.03, representing a slight yellow coloration of the rTA. Specification-compliant values can also be achieved with the activated carbon AC-2 by obtaining a b-value of 0.14. When using the activated carbon AC-3, a color value of 0.21 is reached for the b-value. Even for Amberlite XAD-7HP as adsorbent, a b-value of 1.11 still complies with the specification.

Overall, the color values obtained after adsorption with activated carbon are superior to those achieved using Amberlite XAD-7HP. The more effective decolorization of disperse dyes using activated carbon may be explained by the similarity of polarity between the dye molecules and the activated carbon surface. Disperse dyes are non-polar or weakly polar organic compounds that often contain aromatic or hydrophobic structures. It is hypothesized that these structures interact preferentially with the hydrophobic, non-polar surface of activated carbons. Amberlite XAD-7HP, by contrast, exhibits a slightly polar surface according to manufacturer specifications [42]. Unlike other polymeric resins, Amberlite XAD-7HP is expected to have a low adsorption capacity for disodium terephthalate, which potentially make it an effective option for dye removal.

The presented findings in terms of activated carbon adsorption align with published and previously described data from different research studies [33,43]. Based on the present findings, the results demonstrate that activated carbon adsorption is also applicable for the decolorization of a colored product solution in polyester recycling.

Nevertheless, the interpretation of the results is limited by the unknown chemical structure of the dyes present in the textile waste sample. Typically, the specific dyes present in polyester textile waste are unknown, which necessitates the development of a generic decolorization process. To assess the broader application of the presented results, further studies should systematically investigate the influence of defined dye species on the adsorptive decolorization. Therefore, polyester textiles should be dyed with known dyes from different dye classes. Further adsorption experiments would then provide deeper insights into process optimization and reproducibility.

To elucidate the adsorption process further, investigations on adsorption kinetics should be conducted. Additionally, detailed surface characterization and determination of the point of zero charge are necessary.

Furthermore, additional research is required to investigate the desorption of dye molecules from activated carbon to enable its reuse. Potential structural changes in the dyes may occur during the depolymerization reaction and consequently could influence the adsorption and desorption characteristics. The development of appropriate analytical techniques is required to analyze the dye structures and possible dye degradation products. For later industrial application of the adsorption process, the regenerability of the activated carbon as well as a comprehensive cost analysis must be carefully assessed.

## 4. Conclusions

This article, for the first time, presents the results of adsorptive decolorization of a disodium terephthalate solution from monomer recycling of polyester via alkaline hydrolysis. Activated carbon was found to be an effective adsorbent for the removal of disperse dyes from the aqueous solution. AC-1 appears to be the most suitable activated carbon, as it achieves the best color value for rTA of 2.8 H° after adsorption. For the first time, this study reports optimized adsorption parameters that enable the recycling of rTA in accordance with industry specifications in terms of color value. The adsorption process was shown to be dependent on pH and the temperature of the product solution. The batch studies indicate that a neutral pH value as well as temperatures up to 80 °C are favorable for the adsorption process. Therefore, in industrial application, appropriate heat integration is required to create an economically viable process even at elevated operating temperatures.

Based on the newly presented results, adsorptive decolorization enables the recovery of specification-compliant rTA in terms of color value from polyester recycling. The recovery of high-purity rTA is a key step towards textile-to-textile recycling, thereby contributing to a circular economy of polyester textiles.

## Figures and Tables

**Table 1:** Characteristics of activated carbon.

Activated Carbon Type	Source Material	Activation	Total Surface Area (m^2^/g)	Methylene Blue Adsorption (g/100 g)
AC-1	coconut shell	steam	1400	28
AC-2	coal	steam	1050	23
AC-3	walnut	phosphoric acid	1900	35

**Table 2:** LAB color values of rTA after adsorption compared to the specification for industrial application [30].

Parameter	AC-1	AC-2	AC-3	Amberlite XAD-7HP	Typical Values for Fossil-Based TA	Industrial Specification for TA
L [-]	99.79	99.92	99.89	99.54	95	-
a [-]	−0.01	0.01	0.01	0.1	−0.1	-
b [-]	0.04	0.14	0.21	1.11	1.5–2	<2

## Data Availability

The original contributions presented in this study are included in the article. Further inquiries can be directed to the corresponding author.

## Abbreviations

The following abbreviations are used in this manuscript:

BHET	bis(2-hydroxyethyl)terephthalate
DMT	dimethyl terephthalate
H_2_SO_4_	sulfuric acid
NaOH	sodium hydroxide
Na_2_SO_4_	sodium sulfate
PET	polyethylene terephthalate
rTA	recycled terephthalic acid
TA	terephthalic acid
